# Global Health Implications of Nutrient Changes in Rice Under High Atmospheric Carbon Dioxide

**DOI:** 10.1029/2019GH000188

**Published:** 2019-07-31

**Authors:** M. R. Smith, S. S. Myers

**Affiliations:** ^1^ Department of Environmental Health Harvard T.H. Chan School of Public Health Boston MA USA; ^2^ Harvard University Center for the Environment Cambridge MA USA

**Keywords:** malnutrition, carbon emissions, rice, B vitamins, nutritional inadequacy, birth defects

## Abstract

A growing literature has documented that rising concentrations of carbon dioxide in the atmosphere threaten to reduce the iron, zinc, and protein content of staple food crops including rice, wheat, barley, legumes, maize, and potatoes, potentially creating or worsening global nutritional deficiencies for over a billion people worldwide. A recent study extended these previous nutrient analyses to include B vitamins and showed that, in rice alone, the average loss of major B vitamins (thiamin, riboflavin, and folate) was shown to be 17–30% when grown under higher CO_2_. Here, we employ the EAR cut‐point method, using estimates of national‐level nutrient supplies and requirements, to estimate how B vitamin dietary adequacy may be affected by the CO_2_‐induced loss of nutrients from rice only. Furthermore, we use the global burden of disease comparative risk assessment framework to quantify one small portion of the health burden related to rising deficiency: a higher likelihood of neural tube defects for folate‐deficient mothers. We find that, as a result of this effect alone, risk of folate deficiency could rise by 1.5 percentage points (95% confidence interval: 0.6–2.6), corresponding to 132 million (57–239 million) people. Risk of thiamin deficiency could rise by 0.7 points (0.3–1.1) or 67 million people (30–110 million), and riboflavin deficiency by 0.4 points (0.2–0.6) or 40 million people (22–59 million). Because elevated CO_2_ concentrations are likely to reduce B vitamins in other crops beyond rice, our findings likely represent an underestimate of the impact of anthropogenic CO_2_ emissions on sufficiency of B vitamin intake.

## Key Points

- Global CO_2_ levels that may be reached as soon as 2050 have been shown to lower the B vitamin content of rice by 17–30%
- These losses may cause 132 million people globally to become at risk of deficiency in folate, 67 million in thiamin, and 40 million in riboflavin
- Highly affected regions are those that are mostly nutritionally reliant on rice: Africa (West and East) and Asia (Southeast, East, and South)

## 1 Introduction

Global carbon dioxide emissions continue to rise year over year, producing the highest concentrations of atmospheric CO_2_ ever directly recorded: 405 ppm in 2017 (Dlugokencky & Tans, 2018). Future CO_2_ emission forecasts estimate that we will achieve a global average concentration of 550 ppm CO_2_ in the next 30–80 years, with our current trajectory predicting that we are more likely to reach that level by midcentury (Prather et al., 2013).

The persistent rise in CO_2_ is predicted to have a range of deleterious effects on global food production and nutritional sufficiency (Myers et al., 2017). One specific example that has been demonstrated is the loss of a range of nutrients in food crops when grown under elevated CO_2_. For several decades, multiple experiments examining the response of food crops in controlled indoor environments found significant losses in the concentrations of many elements important for human health (Fangmeier et al., 1997; Manderscheid et al., 1995; Seneweera & Conroy, 1997; Ziska et al., 2004), though these results had been found to be inconclusive when replicated under open‐field free‐air carbon enrichment (FACE) conditions (Lieffering et al., 2004). However, in the past decade, many subsequent FACE experiments have confirmed these earlier results for many nutrients of importance to global health—particularly iron, zinc and protein—and across a range of important staple foods such as rice, wheat, and barley, as well as, for certain nutrients, potatoes, maize, and soybeans (Loladze, 2014; Myers et al., 2014; Taub et al., 2008). Modeling analyses to quantify the ensuing rise in risk of deficiency attributable to these losses of nutrients have shown that 138–175 million people could become newly at risk of zinc deficiency (Myers et al., 2015; Smith & Myers, 2018) and 122–148 million people are newly at risk of protein deficiency in 2050 as a result of this effect (Medek et al., 2017; Smith & Myers, 2018). In addition, over a billion people are likely to experience exacerbations of existing protein and zinc deficiencies. Risk of iron deficiency could not be estimated in a similar way due to a more tenuous link between diet and deficiency, but it was estimated that very large numbers of women in their childbearing years and children under 5 years of age would be at high risk for this effect (Smith et al., 2017). Together, these studies have indicated that CO_2_‐mediated loss of nutrients in staple food crops is a significant threat to global nutritional sufficiency.

A recent study by Zhu et al. (2018) has extended these previous findings by looking more deeply at the impact of higher CO_2_ on both the mineral and vitamin content of rice. This study confirmed the nutritional impacts to iron, zinc, and protein seen in prior studies but also extended their analysis to a range of B vitamins (thiamin, riboflavin, pantothenic acid, and folate) and found even more extreme declines of 13–30% on average. Though this study only explored the implications of a loss of B vitamins from one food, rice, it still illuminates a major vulnerability in our global food system to anthropogenic CO_2_ emissions. On average, rice provides 19% of calories to people globally, and over half of the world derives greater than 25% of their calories from rice each day (Food and Agriculture Organization, FAO, 2019). As such, it is the single most calorically important food globally. Because of the unique importance or rice to global diets, it is likely to play a large role in micronutrient sufficiency as well.

Insufficient intake of the specific B vitamins that are lost under elevated CO_2_ causes a range of health complications (Institute of Medicine, 1998). Thiamin deficiency leads to the disease beriberi, which can result in serious neurologic or cardiovascular symptoms, potentially fatal in serious cases. Riboflavin deficiency can cause anemia, inflammation of the mucosal membranes of the mouth and eyes, and congenital heart defects for those born to riboflavin‐deficient mothers. Inadequate folate intake can result in a higher likelihood of neural tube birth defects due to incomplete closure of the spinal cord during fetal development. In 2010, neural tube defects (NTDs) caused an estimated 48,000 deaths globally and 5.7 million life years lost to death or disability (Global Burden of Disease, GBD, 2018).

In this study, we seek to understand the global health implications of the loss of B vitamins from rice. Using the nutritional content of current diets, paired with predicted losses of nutrition under elevated CO_2_, the national estimated physiologic requirements for each nutrient, and using these as inputs into the EAR cut‐point method to identify the prevalence of inadequate intakes, we examine the increased risk of deficiencies for thiamin, riboflavin, and folate (dietary data are unavailable for vitamin B5, pantothenic acid). Despite the potentially broad public health consequences across all B vitamins, we also attempt to quantify the size of the burden of disease related to one specific outcome with an established risk‐disease relationship using the established global burden of disease comparative risk assessment framework: increased risk of NTDs for mothers with inadequate folate intakes. Understanding that this outcome represents only a small piece of the broader health implications of this effect, our goal is to at least partially grasp the scale and geographic distribution of those health implications for which we have sufficient data to measure.

## 2 Materials and Methods

### 2.1 Nutrient Supplies by Age, Sex, and Country in 2010

Per capita nutrient supplies for folate, thiamin, and riboflavin were collected from the publicly available Global Expanded Nutrient Supply (GENuS) data sets (Smith, 2016), whose composition and construction has been described (Smith et al., 2016). Nutrient supply data are provided for each food in the diet, and for 5‐year age‐sex bins in each country for 2010. We use nutrient supplies as an estimate for nutrient intake, an assumption that has accompanying caveats as provided in the discussion section below.

The additional contribution of fortification was also taken into account as it provides a large component of these particular nutrients in many developed countries. Here, we used estimates of the fortification levels for wheat flour, rice, and maize flour for each nutrient as provided by the Food Fortification Initiative (FFI, 2018a). Based on the commercial form of fortificant used in each country, the amount was converted to its equivalent of the pure nutrient using standardization factors collected from Ottaway (1993). Where multiple possible fortificants were listed in the FFI table, their average standardization value was used. These fortification levels were then paired with FFI's country estimates of the percentage of each food that is industrially processed, and also the proportion of industrially processed grains that are assumed to be fortified; multiplying these two values together yields the amount of each grain and flour that is assumed to be fortified in each country.

### 2.2 Physiological Requirements

The recommended nutrient intakes for each nutrient (FAO/World Health Organization, WHO, 2004) were calculated using weighted averages to harmonize their age groupings with the 5‐year age bins in GENuS. These were then converted to estimated average requirements (EARs) using conversion factors (Allen et al., 2006).

For each nutrient, requirements are generally higher for pregnant and lactating women. To estimate the population size of both, we used age‐specific fertility rates from the United Nations World Population Division (2018). The number of pregnant women is estimated by multiplying the annual birthrate by the proportion of the year that constitutes an average pregnancy: 40 weeks/52 weeks. The number of lactating women was estimated by assembling a database on the average duration of breastfeeding by mothers in each country (World Breastfeeding Trends Initiative, 2018; WHO, 2018) also multiplied by the birth rate. Regional averages were used for countries without data. Knowing the number of pregnant and lactating women in each age‐sex group then allowed us to construct a population‐weighted EAR for each age group for women between the age of 15 and 49.

### 2.3 Populations Newly at Risk of Deficiency Under Current Conditions and Elevated CO_2_

Using the availability of each nutrient and the national EARs, we then sought to estimate the proportion of the population that would be placed newly at risk of deficiency in certain B vitamins as a result of losing nutrients from rice due to elevated CO_2_ levels.

To achieve this, we first needed to determine the populations currently consuming insufficient amounts of each nutrient compared to their physiologic needs. The customary way of performing this calculation at the population level is the EAR cut‐point method (Institute of Medicine, 2000), which has been used in numerous studies to investigate similar questions of population‐level nutritional adequacy (Arsenault et al., 2015; Beal et al., 2017; Joy et al., 2014; Medek et al., 2017; Myers et al., 2015; Smith et al., 2017; Wessells & Brown, 2012). As inputs, the EAR cut‐point method requires a distribution of intakes, as well as a single EAR value for each group of interest. The EAR was estimated using the steps outlined in section 2.2, whereas additional steps were required to construct intake distributions, because GENuS merely provides average nutrient supplies rather than distributions. To construct intake distributions from national means, we follow the convention of previous studies and assume a normal distribution around the mean with a coefficient of variation of 30% for B vitamins based on empirical evidence (Arsenault et al., 2015; Beal et al., 2017). Combining intakes and requirements using the EAR cut‐point method, we then are able to calculate the proportion of the population which is consuming an inadequate amount of each nutrient, which is equivalent to the proportion consuming less than the EAR, given that the requirements to use the cut‐point method are met for these nutrients.

Next, we estimated how elevated CO_2_, and its consequences for the nutritional content of rice only, may affect the proportion of each country at risk of deficiency in B vitamins. To do so, we used the relationships between eCO_2_ and losses to B vitamin content in rice established by Zhu et al. (2018). Zhu et al. measured the nutritional response of rice grown under eCO_2_ in 18 cultivars meant to capture the mostly widely cultivated lines globally, including both varietal groups (Indica and Japonica) as well as newer hybrid lines. Paired samples for each cultivar were grown under identical environmental conditions and management levels (fertilizer and pesticide use), except for that one was grown under elevated ambient CO_2_ levels of ~580 ppm using FACE experimental protocols. Grain samples were then harvested, analyzed and compared, and relationships in the vitamin content between them was established including associated uncertainties. Using these relationships, we combined the modeled losses of nutrients with the average total dietary supplies for each nutrient from GENuS to estimate the nutrients supplied by prefortified rice before and after the CO_2_‐related decline in nutritional content. Additional nutrients from fortification were then added to both the values representing the ambient and elevated CO_2_ cases. The ratio of these two values was then multiplied by the current intake distribution to estimate the CO_2_‐impacted intake distribution. Once again using the EAR cut‐point method, the proportion of the population consuming in inadequate amount of each nutrient under eCO_2_ was then calculated, and the difference between current and elevated CO_2_ conditions was determined.

Finally, to ascertain the population size potentially affected in 2050, current and eCO_2_‐affected proportions at risk of deficiency was multiplied by the population size of each age‐sex group in every country in 2050 (UN World Population Prospects, 2017).

### 2.4 Increases in Mortality and Morbidity

Due to the relationship between folate intake and the incidence of developing fetal NTDs, we sought to estimate the public health consequences of the increased risk of folate deficiency in terms of potential lives and life years lost to death and disability.

To do this, we used the standard global burden of disease comparative risk assessment framework (Murray et al., 2003) and current burden of NTDs (GBD, 2018) to estimate the risk‐attributable burden from CO_2_‐induced increases in folate deficiency. This was performed by isolating the incremental risk of reduced folate intake, called the potential impact fraction (PIF). The PIF is calculated as the incremental percentage increase in the exposure‐weighted risk of incidence of NTDs under higher CO_2_ relative to current conditions. Relative risks were estimated as a best fit polynomial curve through the folate‐NTD data set from the Institute of Medicine (1998) and the folate intake distributions of pregnant women were used as exposure levels. The PIF for each country was multiplied by the 2010 burden of deaths and disability‐adjusted life years (DALYs) caused by NTDs in each country (GBD, 2018). The resulting values represent the incremental rise in deaths and DALYs attributable to higher CO_2_ and its effect on the nutritional content of rice.

### 2.5 Treatment of Uncertainty

Most of the inputs used come with associated uncertainties: food intake by age and sex, nutrients from the diet, the effect of CO_2_ on nutritional density of rice, and deaths and DALYs lost to NTDs. GENuS data sets of food and nutrient availabilities were generated with associated uncertainties derived from Monte Carlo modeling to capture the uncertainty within the age‐sex‐specific intakes of foods, as well as the potential nutrient density of each food based on the range of possible candidates that may adequately describe each food. Full characterization of uncertainty in GENuS may be found in Smith et al. (2016).

For two other inputs—the effect of CO_2_ on the nutrient content of rice and the current burden of disease related to NTDs—results were previously reported as means with 95% confidence intervals. Using these summary statistics, we generated skew‐normal distributions to match these parameters, and then used these distributions as inputs into our own Monte Carlo simulations for these studies.

Despite our efforts to characterize the full uncertainty in our model, some of our inputs do not have measured and reported uncertainties, specifically the food supplies provided by the UN‐FAO and the relative risks of NTDs for children born to folate‐deficient mothers. Therefore, our uncertainty estimates are likely smaller than their true value, though we are unable to fully characterize their true size.

Regardless of these limitations, we still aim to generate estimates of the uncertainties for our results to the greatest extent possible using Monte Carlo simulations (*N* = 1,000). For each of our uncertainty distributions associated with our inputs, we randomly sample from among them, perform the calculations described above to generate our estimates, and then select the middle 95% values as uncertainty intervals.

## 3 Results

Under elevated CO_2_, loss of nutrition from rice could contribute to the increase of those at risk of folate deficiency by an additional 1.5 percentage points (95% uncertainty interval: 0.6–2.6), as well as 0.7 points (0.3–1.1) for thiamin and 0.4 points (0.2–0.6) for riboflavin (Table 1). These percentages equate to an additional 132 million people (57–239M) potentially becoming newly at risk of folate deficiency in 2050, 67 million people (30–110M) newly at risk of thiamin deficiency, and 40 million people (22–59M) newly at risk of riboflavin deficiency. In addition, we anticipate exacerbations of health burdens for those already consuming inadequate amounts of each nutrient for a much larger proportion of the global population in 2050: roughly 2.5 billion people (2.2–2.9B) for folate, 3.4 billion people (3.0–3.8B) for thiamin, and 2.8 billion people (2.6–3.1B) for riboflavin.

**Table 1:** Contribution of Rice to Selected B Vitamins and Prevalence of Those at Risk of Deficiency (Current and Estimated Under Elevated CO_2_), by Geographic Region

Region name	2010 Population (millions)	Current prevalence of inadequate intake (%)			Contribution of rice to total dietary supply (%)			Increase in risk of deficiency under eCO_2_ (percentage points)			Increase in population newly at risk of deficiency under eCO_2_ (millions)		
		Folate	Thiamin	Riboflavin	Folate	Thiamin	Riboflavin	Folate	Thiamin	Riboflavin	Folate	Thiamin	Riboflavin
Eastern Africa	322	8.3 (4.7–11.6)	32.3 (26.2–39.2)	24.8 (14.3–28.1)	2.9 (2.5–4.9)	3.2 (2.2–9.2)	3.2 (1.4–4.7)	0.6 (0.3–1.2)	0.5 (0.2–0.9)	0.4 (0.2–0.7)	5.6 (2.7–10.6)	4.1 (1.3–7.7)	3.1 (1.7–6.1)
Middle Africa	66	6.9 (5.1–9.8)	34.7 (29.5–40.6)	19.5 (14.8–23.9)	2.2 (1.7–3.6)	2.6 (1.3–7.7)	2.4 (1–3.6)	0.2 (0.1–0.8)	0.4 (0.2–0.7)	0.2 (0.1–0.4)	0.4 (0.1–1.3)	0.7 (0.3–1.3)	0.3 (0.2–0.7)
Northern Africa	204	7.6 (5.5–10.5)	14.7 (5.5–22.7)	20.8 (17.9–23.5)	1 (1–1.1)	7.1 (6.5–16.2)	2.2 (1.6–2.7)	0.8 (0.4–1.4)	0.2 (0.1–0.4)	0.1 (0.1–0.2)	2.9 (1.4–4.8)	0.8 (0.2–1.5)	0.4 (0.2–0.6)
Southern Africa	59	5.1 (3.2–8)	10.3 (6.5–18.2)	18 (11.1–21.9)	3.6 (3.1–7.1)	1.9 (1.4–9.3)	2 (0.8–3.4)	0.2 (0–0.7)	0.1 (0–0.3)	0.2 (0.1–0.4)	0.1 (0–0.6)	0.1 (0–0.2)	0.1 (0.1–0.3)
Western Africa	308	5.1 (2.1–10.3)	20 (14.8–27.4)	8.2 (6.4–10.3)	6.4 (5.7–11.2)	6.2 (4–21.8)	7.1 (3.1–10.5)	0.7 (0.3–1.6)	0.7 (0.3–1.4)	0.4 (0.2–0.7)	5.5 (2.5–13.1)	5.9 (2.3–11.3)	2.9 (1.6–5.6)
Northern America	343	6.7 (4.1–12.6)	2.6 (0.8–13.6)	2.7 (2.2–3.1)	0.4 (0.4–0.5)	2.7 (2.1–8)	0.5 (0.3–0.8)	0.3 (0.1–1)	0 (0–0.1)	0 (0–0)	1.4 (0.3–4.6)	0 (0–0.3)	0 (0–0)
Central America	161	3.3 (0.7–13.5)	6.3 (1.1–17.3)	7.6 (5–9.4)	0.6 (0.5–0.7)	1.7 (0.6–6.6)	1 (0.2–2)	0.1 (0–0.5)	0 (0–0.2)	0 (0–0)	0.1 (0–1.3)	0.1 (0–0.4)	0 (0–0.1)
Caribbean	36	7.3 (1.2–19.8)	26 (10.3–41.5)	22.9 (18.4–25.7)	2.1 (1.9–2.3)	10.7 (4.5–29.6)	6.4 (1.6–11)	0.9 (0.1–3.6)	0.6 (0.1–1.5)	0.2 (0.1–0.3)	0.5 (0–1.9)	0.3 (0.1–0.8)	0.1 (0.1–0.2)
South America	395	7.3 (0.6–22.9)	9.2 (0.8–26)	9.6 (7.4–11)	1.5 (1.3–1.6)	4.8 (1.4–18.7)	2.4 (0.5–5.3)	0.5 (0–3)	0.1 (0–0.7)	0.1 (0–0.1)	2.7 (0.1–16.4)	0.8 (0–3.9)	0.4 (0.2–0.6)
Central Asia	63	22.7 (14.7–32.6)	18.9 (5.3–46.2)	46 (28.9–56)	0.5 (0.4–0.6)	4.2 (2.6–8.8)	0.7 (0.4–1)	1 (0.4–2.4)	0.1 (0–0.2)	0 (0–0.1)	1 (0.4–2.3)	0.1 (0–0.1)	0 (0–0.1)
Eastern Asia	1565	12.9 (7.5–16)	14.9 (6.1–28.8)	15.8 (6.5–26.3)	3.1 (2–3.6)	13.5 (10.8–14.8)	5.9 (3.8–8.2)	2 (0.8–3.4)	0.5 (0.1–1.3)	0.2 (0.1–0.6)	31.1 (12.6–53.9)	8.4 (2–21.7)	3.9 (0.9–9.4)
Southern Asia	1705	75.5 (74.4–80.4)	83.6 (80.5–86.4)	47.6 (42.3–53.4)	5.8 (5.5–6.1)	6.1 (2.9–7.9)	3.6 (1.8–5.2)	0.8 (0.2–1.9)	0.6 (0.1–1.1)	0.8 (0.4–1.2)	17.6 (4.5–43.5)	14.4 (2.7–26.7)	19.5 (10.5–28.9)
South‐Eastern Asia	592	17.8 (4–49.5)	43.3 (26.3–65.5)	50.8 (39.6–57.2)	9.9 (8.8–10.7)	45.8 (20.5–65.5)	22.6 (9–34.2)	6.9 (2.4–13.6)	3.6 (1.4–6.4)	1 (0.6–1.5)	56.2 (19.4–112.1)	29.9 (11.3–52.8)	8.1 (4.4–11.8)
Western Asia	227	1.8 (1.3–2.4)	11.4 (5.5–15.7)	18.3 (16.3–20.4)	0.9 (0.9–1)	3.7 (3.5–10.3)	1.7 (1.4–2.1)	0.1 (0.1–0.3)	0.2 (0.1–0.3)	0.1 (0.1–0.1)	0.6 (0.2–1.2)	0.9 (0.3–1.3)	0.4 (0.2–0.6)
Eastern Europe	295	11.4 (7.9–17.5)	8.7 (3.6–24)	58.6 (40.7–74)	0.2 (0.2–0.3)	1.4 (1.1–4.2)	0.3 (0.2–0.4)	0.3 (0.1–0.8)	0 (0–0.1)	0 (0–0.1)	0.7 (0.2–2.2)	0 (0–0.2)	0.1 (0.1–0.2)
Northern Europe	100	5 (3.2–7.9)	5.4 (1.9–17.4)	52.9 (43.1–64.8)	0.4 (0.3–0.4)	2.1 (1.7–6.3)	0.4 (0.3–0.6)	0.1 (0–0.5)	0 (0–0.1)	0.1 (0–0.1)	0.2 (0–0.6)	0 (0–0.1)	0.1 (0–0.1)
Southern Europe	154	7.5 (5.2–10.8)	6.3 (2.8–14.1)	35.3 (27.9–44)	0.4 (0.3–0.4)	2.2 (1.9–6.5)	0.5 (0.4–0.6)	0.2 (0.1–0.6)	0 (0–0)	0.1 (0–0.1)	0.3 (0.1–1)	0 (0–0.1)	0.1 (0–0.1)
Western Europe	188	7.4 (4.1–12.2)	3.6 (1.1–10.4)	53.2 (40.2–67.3)	0.3 (0.2–0.3)	1.5 (1.1–4.4)	0.3 (0.2–0.4)	0.2 (0–0.6)	0 (0–0)	0 (0–0.1)	0.4 (0.1–1.3)	0 (0–0.1)	0.1 (0–0.1)
Australia and New Zealand	26	3 (1.7–5.1)	2 (0.7–9.4)	6.1 (4.5–8.2)	0.7 (0.6–0.7)	4.1 (2.9–11.6)	0.6 (0.4–0.9)	0.1 (0–0.5)	0 (0–0.1)	0 (0–0)	0.1 (0–0.2)	0 (0–0)	0 (0–0)
Melanesia	2	16.6 (12.2–22.1)	39 (30.3–47.2)	20.9 (14–25.5)	2.9 (2.4–3.5)	25.7 (14.7–37.5)	7.2 (6.1–8.3)	2.2 (0.9–4)	1 (0.6–1.5)	0.1 (0–0.2)	0.1 (0–0.1)	0 (0–0)	0 (0–0)
Micronesia	0.1	9.4 (2.7–41.8)	66.8 (19.7–91.9)	74.6 (1.1–93.8)	5.3 (1.3–6.9)	39.7 (19–58)	10.8 (7–14.4)	3.7 (0.9–11.5)	1.6 (0.6–2.3)	0.5 (0–0.9)	0 (0–0)	0 (0–0)	0 (0–0)
Polynesia	0.5	45.8 (42.4–49.5)	54 (47.9–61.2)	68.9 (53.3–74)	2.1 (1.5–2.3)	14.7 (9.5–26.4)	3.1 (2.5–3.8)	1.1 (0.4–2)	0.2 (0.1–0.4)	0.1 (0.1–0.2)	0 (0–0)	0 (0–0)	0 (0–0)
Global	6811	26.7 (24.1–31)	34.2 (29.9–39.2)	29.9 (26.5–33.4)	3.8 (3.4–4.2)	11.1 (7.4–14.1)	5.2 (3.3–6.8)	1.5 (0.6–2.6)	0.7 (0.3–1.1)	0.4 (0.2–0.6)	132.1 (56.8–238.8)	66.6 (30.2–110.3)	40.4 (21.8–59.3)

Quantifying one small piece of the health burden that could arise from this increase in those at risk of deficiency, we find that the health burden attributable to an increased risk of NTDs associated with the rise in folate deficiency would roughly translate to an additional 27,900 life years lost annually (13,700–56,800; Table 2). Furthermore, this impact of rising folate inadequacy could cause roughly an additional 260 (130–590) deaths annually from NTDs. Both the increase in deaths and life years lost represent an increase of 0.5% over current rates.

**Table 2:** Burden of Disease From Neural Tube Defects, Current, and Estimated Attributable to Loss of Folate From Rice Under Elevated CO_2_

WHO microregion name	2010 population (millions)	Current burden of neural tube defects (annual rate per million people)		Current burden of neural tube defects (total number per year)		Additional burden of NTDs with eCO_2_ (rate per million people)		Additional burden of NTDs with eCO_2_ (total number per year)	
		DALYs lost	Deaths	DALYs lost (000)	Deaths	DALYs lost	Deaths	DALYs lost (000)	Deaths
Eastern Africa	322	1582 (1244–2064)	14.6 (10.8–19.9)	509.3 (400.6–664.4)	4692 (3464–6403)	6.8 (3.1–16.4)	0.07 (0.03–0.15)	2.2 (1–5.3)	22 (10–49)
Middle Africa	66	5180 (3598–7162)	55 (37.2–76.7)	341.3 (237–471.8)	3624 (2448–5050)	17.9 (7.7–45.9)	0.18 (0.08–0.52)	1.2 (0.5–3)	12 (5–34)
Northern Africa	204	1547 (1078–2278)	12.1 (6.9–20.3)	315.3 (219.8–464.2)	2465 (1398–4136)	0.8 (0.4–1.4)	0.01 (0–0.01)	0.2 (0.1–0.3)	1 (0–2)
Southern Africa	59	578 (487–683)	3.4 (2.9–4)	34.1 (28.7–40.3)	199 (170–236)	0.8 (0.4–1.8)	0 (0–0.01)	0 (0–0.1)	0 (0–1)
Western Africa	308	6041 (4142–8759)	64.4 (43.4–98.2)	1859.2 (1274.7–2695.9)	19826 (13366–30221)	50.6 (21.7–128.8)	0.54 (0.24–1.46)	15.6 (6.7–39.6)	166 (73–450)
Northern America	343	266 (225–310)	1.5 (1.3–1.6)	91.1 (77.2–106.1)	514 (437–565)	0.2 (0.1–0.4)	0 (0–0)	0.1 (0–0.1)	0 (0–1)
Central America	161	782 (691–886)	5.7 (5.1–6.5)	125.5 (110.9–142.3)	918 (822–1049)	0.4 (0.2–0.7)	0 (0–0.01)	0.1 (0–0.1)	0 (0–1)
Caribbean	36	1145 (621–1970)	12.4 (6–21.2)	41.8 (22.7–71.9)	451 (220–776)	1.5 (0.7–2.8)	0.01 (0.01–0.03)	0.1 (0–0.1)	1 (0–1)
South America	395	695 (625–783)	5.6 (4.9–6.5)	274.5 (247–309.2)	2203 (1955–2551)	2.4 (1.2–3.6)	0.02 (0.01–0.03)	0.9 (0.5–1.4)	8 (4–12)
Central Asia	63	974 (768–1226)	7.3 (5.1–10)	61.5 (48.5–77.4)	461 (320–631)	0.5 (0.3–0.9)	0 (0–0.01)	0 (0–0.1)	0 (0–0)
Eastern Asia	1565	150 (125–176)	0.6 (0.6–0.8)	235.3 (195–276.1)	988 (872–1183)	0.3 (0.2–1.7)	0 (0–0.01)	0.5 (0.2–2.7)	2 (1–11)
Southern Asia	1705	266 (225–310)	2.6 (1.8–3.9)	808.5 (644.4–1008.3)	4434 (3015–6599)	1.7 (0.9–2.6)	0.01 (0–0.01)	2.9 (1.5–4.5)	12 (6–20)
South‐Eastern Asia	592	818 (671–971)	7.2 (5.5–9)	484.7 (397.2–574.8)	4263 (3265–5317)	5.2 (2.7–8.2)	0.04 (0.02–0.08)	3.1 (1.6–4.9)	27 (14–45)
Western Asia	227	1220 (957–1582)	8.9 (6–12.8)	276.5 (217–358.7)	2028 (1356–2904)	0.8 (0.4–1.3)	0.01 (0–0.01)	0.2 (0.1–0.3)	1 (1–2)
Eastern Europe	295	322 (277–363)	1.3 (1.1–1.7)	94.8 (81.7–106.9)	398 (321–502)	0.1 (0–0.1)	0 (0–0)	0 (0–0)	0 (0–0)
Northern Europe	100	228 (208–251)	1 (0.9–1.1)	22.8 (20.8–25.1)	104 (91–114)	0.1 (0–0.1)	0 (0–0)	0 (0–0)	0 (0–0)
Southern Europe	154	180 (161–201)	0.4 (0.4–0.5)	27.6 (24.7–30.8)	67 (56–77)	0 (0–0.1)	0 (0–0)	0 (0–0)	0 (0–0)
Western Europe	188	175 (148–205)	0.5 (0.4–0.5)	32.9 (27.9–38.6)	88 (72–101)	0 (0–0)	0 (0–0)	0 (0–0)	0 (0–0)
Australia and New Zealand	26	259 (212–306)	1.3 (1–1.5)	6.9 (5.6–8.1)	34 (27–41)	0.4 (0.2–0.5)	0 (0–0)	0 (0–0)	0 (0–0)
Melanesia	2	820 (539–1217)	8.3 (4.9–12.7)	1.5 (1–2.3)	16 (9–24)	3 (1.5–5.2)	0.03 (0.01–0.05)	0 (0–0)	0 (0–0)
Micronesia	0.1	516 (275–875)	4.5 (1.5–8.2)	0.1 (0–0.1)	0 (0–1)	2.2 (0.7–6.7)	0.02 (0–0.06)	0 (0–0)	0 (0–0)
Polynesia	0.5	152 (93–262)	1 (0.5–2.3)	0.1 (0–0.1)	0 (0–1)	0.1 (0–0.2)	0 (0–0)	0 (0–0)	0 (0–0)
Global	6811	834 (733–966)	7.1 (6–8.7)	5681.9 (4994.8–6579.1)	48305 (40884–59177)	4.1 (2–8.3)	0.04 (0.02–0.09)	27.9 (13.7–56.8)	261 (127–591)

*Note*. NTD = neural tube defect; DALY = disability‐adjusted life year; WHO = World Health Organization.

**Figure 1:**
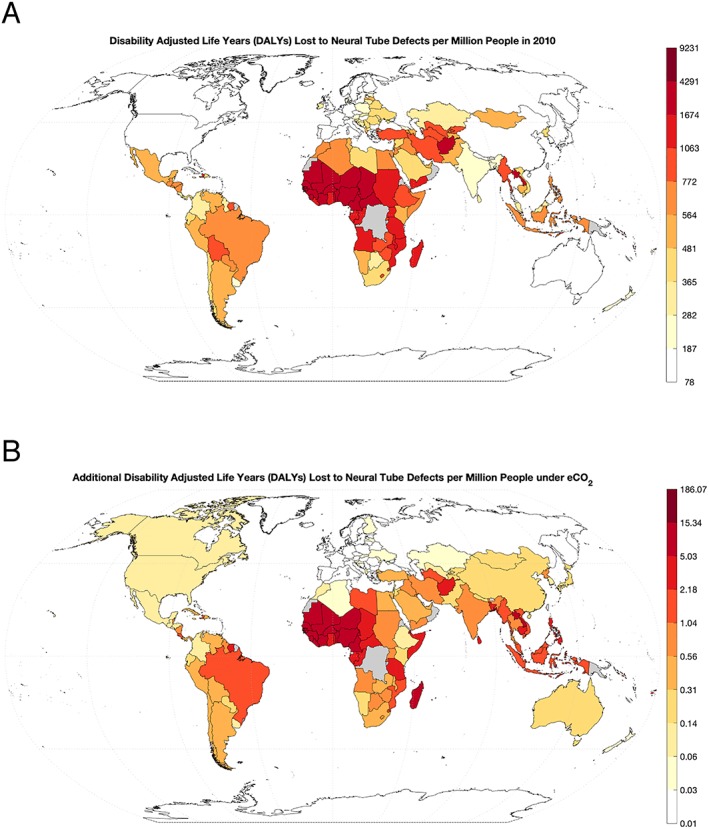
Global rate of disability‐adjusted life years lost to neural tube defects in (a) 2010 and (b) modeled under elevated CO_2_ conditions.

The geographic distribution of the burden of increased risk of deficiency from vitamin B losses in rice falls most heavily on regions that are reliant on rice for nutrition and are currently suffering higher rates of inadequacy in the B vitamins studied: South, East, and Southeast Asia and Oceania, and to a lesser degree parts of Western and Eastern Africa (Table 1). This pattern also translates to the health burden of folate‐deficiency‐related NTDs, though these would fall more heavily on those regions that already struggle from a higher incidence of NTDs: mainly Western, Central, and Eastern Africa, as well as Southeast Asia (Figure 1).

However, the predicted growth in the deaths and life years lost associated with increased risks of folate deficiency is lower than the rise in those newly at risk of folate deficiency overall. This is due to a slight mismatch between the locations suffering the largest rises in deficiency risks under higher CO_2_ and those that are currently enduring the highest burdens of disease from NTDs. Africa currently has the highest rates of NTDs globally, though is predicted to only experience middling growth in folate inadequacy under elevated CO_2_, whereas much of Asia experiences the opposite situation. As a result, the health consequences of rising risks of folate deficiency are dampened by geographically disparate impacts.

## 4 Discussion

These results suggest that higher CO_2_ could produce a large increase in the size of the population at risk for deficiency in thiamin, riboflavin, and folate while exacerbating existing deficiencies for many more. On the other hand, we also find that the public health burden for the only quantifiable outcome related to these changes in nutrient intake—folate and NTDs—is somewhat modest by comparison (an 0.5% increase in both deaths and DALYs). Though this study focuses on the limited case of the global health implications from a single health outcome attributable to the loss of certain B vitamins from rice, it is a very conservative estimate of the public health burden of the effect of elevated CO_2_ on the nutritional content of food. As previous studies have shown, most other micronutrients also tend to show a significant loss for many major agricultural crops, indicating a broader and more substantial effect than that presented here. For example, rice contributes to 3.8% of global dietary folate (ignoring fortification), the fifth highest of all foods. Of the other 10 highest‐contributing foods that collectively constitute just over 50% of the dietary folate, nine of these (wheat flour, wheat, fresh vegetables not elsewhere specified, beans, soybeans, potatoes, chickpeas, cabbage and other brassicas, and groundnuts, in descending order) all belong to categories that have shown demonstrable losses of other nutrients under elevated CO_2_ in previous studies. The final food, chicken eggs, is the only one that is derived from an animal source food that has shown no direct measured effect. Therefore, if the loss of B vitamins were as widespread among other foods as that of iron, zinc, or protein, the health implications could be considerably more severe than estimated here.

Furthermore, because we only examine one health outcome with a quantifiable relationship between nutrition and disease—folate and NTDs—our estimate likely represents a very small portion of the potential health effects. Folate deficiency can affect physiological function of all ages and sexes, and the growth of the thiamin‐ and riboflavin‐inadequate population could likewise have population‐level consequences for each. Likely more severe, lowering the nutrient intake for whole populations could deepen the deficiencies that already exist among billions of people globally, imperiling the health of the suffering even further. On the other hand, deaths and life years lost attributable to NTDs have fallen by nearly half since 1990 (GBD, 2018), likely caused by an increase in prenatal screenings, folate supplementation, and fortification programs (Olney & Mulinare, 2002). As such, the impact of slight increases in those at risk of dietary folate deficiency are likely to have a declining impact on the health burden attributable to NTDs in the ensuing decades.

Our study comes with many sources of uncertainty that may impact these estimates. The first, and most pronounced, is our assumption of constant diets to 2050. Here, we have adopted the approach of many previous studies (Medek et al., 2017; Myers et al., 2015; Smith et al., 2017; Smith & Myers, 2018) based on their same rationale. Estimates of future diets are subject to great uncertainty, with most predicated on economic growth projections that inform the overall quantity and mix of foods consumed. However, great uncertainty remains in our ability to predict the course of economic growth, as well as climate change and the consequences of our ongoing global exploitation of natural resources. As a result, both the sustained global economic growth projections and our ability to produce sufficient food to meet economically determined diet estimates for a rapidly growing human population are in doubt. In the face of such uncertainty, we adopt the approach of keeping diets constant not as a prediction of the future, but as a transparent and simple assumption.

In addition, there are other unquantifiable uncertainties in the data sources used that could influence our estimates. Per capita food and nutrition supplies drawn from GENuS are based on the UN‐FAO food balance sheets, as well as from additional FAO production and trade data sets. Because these estimates are made using national‐level reporting of the flows of major commodities rather than household‐ or individual‐level surveys of diet, it has been previously shown that they may be imperfect recorders of individual‐level dietary patterns by neglecting within‐home nutrient loss through processing, cooking, preparation, or food waste (Del Gobbo et al., 2015; Serra‐Majem et al., 2003). As a result, these data are potentially overestimates of true intake, and our estimates of the growth in the population at risk of deficiency should be treated as underestimates of the true values.

Finally, we are also unable to quantify the coverage and dosage of vitamin or dietary supplement intake, in particular folic acid supplementation among women of child‐bearing age. In many developed countries, this could constitute a large increase in folate intake over dietary sources alone. However, these are likely to be less prevalent in many of the developing countries that shoulder much of the burden of increased risks of deficiency under elevated CO_2_, and we therefore assume that this exerts a negligible impact on our estimates.

There are many potential solutions to help avoid these harmful health consequences, though they often require large investments of material resources or government‐level interventions to execute: national supplementation or fortification programs, biofortification of crops, and initiatives to increase dietary diversity and promotion of nutrient‐rich food groups. However, in the case of fortification, the per‐person cost may end up being quite small. A simple illustration of the potential incremental cost of rice fortification: if we assume that fortifying rice with folic acid generally costs $10–30 per ton (FFI, 2018b) and that folic acid fortification rate would continue at the global average of 1.5 mg/kg (FFI, 2018a), making up for the loss in folate consumption induced by higher CO_2_ levels of between 0 and 11 mcg per person per day would cost at most $0.03 USD per year. However, this price only represents the incremental cost of increasing existing fortification programs to offset eCO_2_‐related losses. All countries except nine (Bangladesh, Costa Rica, India, Nicaragua, Panama, Papua New Guinea, Philippines, USA, and Venezuela; FFI, 2018a) do not fortify rice, and starting a new national fortification program is accompanied with significant start‐up investment costs in fortification technology, planning, and outreach. However, these costs remain poorly understood and quantified, so fully encapsulating the costs as part of this hypothetical example is not yet possible.

Another more direct tactic to address the issue here would be to act concertedly to curb CO_2_ emissions growth. However, in addition to these large‐scale solutions, the most recent paper by Zhu et al. (2018) highlights a unique opportunity of rice to solve this problem. Rice cultivars exhibit a remarkable diversity of nutritional content under elevated CO_2_, with some strains showing little or no nutritional response to this effect. This unlocks a potential path toward selective breeding among these resistant cultivars to incorporate this trait as a priority in future breeding programs.

## 5 Conclusions

Here, we demonstrate that the loss of nutrients from one important food, rice, under elevated CO_2_ may have large implications for global health. Hundreds of millions of people who rely upon rice for nutrition may become newly at risk for deficiency in B vitamins as a result of rising CO_2_, causing potentially severe health consequences predominantly for many living in developing countries in Africa and Asia. These results offer a small slice of the broad implications for the impact of higher CO_2_ on nutrition and health. Taken together, CO_2_‐induced nutritional declines could produce a major headwind on progress toward alleviating malnutrition and deserves attention and concerted action.

## Conflict of Interest

The authors declare no conflicts of interest relevant to this study.
